# Comparative Outcomes Between Cruciate-Retaining and Posterior-Stabilized Prostheses in Total Knee Arthroplasty

**DOI:** 10.7759/cureus.87465

**Published:** 2025-07-07

**Authors:** Edgar Iván García-Estrada, Emmanuel Ramírez-Yañez, Gilberto Álvarez-Alarcón, David Muñoz-Nieto, José Juan Villaseñor-Valdés, Luis Antonio Núñez-García

**Affiliations:** 1 Orthopedic Surgery, Hospital de Especialidades Instituto Mexicano del Seguro Social (IMSS) Bienestar, Tampico, MEX; 2 Orthopedic Surgery, Hospital General de Zona of Instituto Mexicano del Seguro Social (IMSS) 7, Monclova, MEX

**Keywords:** longitudinal, patient-reported outcome measures, posterior cruciate retaining prostheses, posterior stabilized prostheses, total knee arthroplasty

## Abstract

Introduction

Total knee arthroplasty (TKA) is the definitive surgical treatment for advanced gonarthrosis when conservative measures fail. Among the available implant designs, posterior cruciate-retaining (PCR) and posterior-stabilized (PS) prostheses are widely used, yet their comparative performance in long-term functional recovery and health-related quality of life remains under debate.

Methods

We conducted a prospective observational study involving 48 elderly patients undergoing primary TKA at a tertiary-care center in Mexico. Participants received either PCR (n=27) or PS (n=21) implants based on intraoperative assessment. Patient-reported outcomes were evaluated using the Western Ontario and McMaster Universities Osteoarthritis (WOMAC) Index, the 36-Item Short Form Health Survey (SF-36), and Patient-Reported Outcomes Measurement Information System (PROMIS-10) Global Health questionnaire at preoperative, six, 12, and 24 months postoperatively.

Results

Both groups demonstrated improvements in all outcome measures; however, PCR patients consistently achieved better results across the follow-up period. At 24 months, WOMAC scores, which reflect better symptom control with higher values, were superior in the PCR group for pain (77.5 vs. 71.0), stiffness (72.5 vs. 62.5), and physical function (86.5 vs. 82.6). SF-36 also favored PCR in physical functioning (66.5 vs. 64.1), bodily pain (72.1 vs. 70.0), and role-physical (54.0 vs. 51.0). PROMIS-10 confirmed these trends, with higher physical (73.9 vs. 71.3) and mental health scores (75.2 vs. 72.0) among PCR patients at 24 months.

Conclusions

Patients receiving cruciate-retaining prostheses experienced greater and more sustained improvements in pain relief, joint function, and quality of life compared to those with posterior-stabilized implants. These findings support the functional advantage of PCR designs in elderly patients undergoing TKA and highlight the value of long-term, multidimensional outcome assessment using patient-reported outcome measures (PROMs).

## Introduction

Knee osteoarthritis (OA) is the most prevalent degenerative joint disorder worldwide, particularly affecting individuals over the age of 60, with a reported prevalence of 10% in men and 13% in women [1]. In Mexico, OA constitutes a major public health concern, accounting for more than 1.5 million annual consultations at the Instituto Mexicano del Seguro Social (IMSS) [2]. As the disease progresses, it leads to chronic pain, stiffness, and loss of function, significantly impairing mobility and quality of life [3]. When conservative management fails, total knee arthroplasty (TKA) remains the gold-standard intervention for symptom relief and functional restoration. Among the prosthetic options, posterior cruciate-retaining (PCR) and posterior-stabilized (PS) designs are widely used; however, the evidence remains inconclusive regarding their comparative benefits in terms of functional recovery and patient satisfaction [4]. In recent years, patient-reported outcome measures (PROMs) have gained prominence as standardized tools to assess clinical effectiveness from the patient’s perspective, focusing on pain, physical performance, and health-related quality of life [5]. In this context, the present study aimed to compare PCR and PS prostheses in elderly patients undergoing TKA, evaluating differences in recovery trajectories and patient-perceived outcomes.

## Materials and methods

We conducted a prospective longitudinal study at the IMSS-Bienestar Specialty Hospital “Dr. Carlos Canseco,” including patients who underwent primary total knee arthroplasty (TKA) in October 2022 (Protocol No. HGT/IMSS-4534-2023). Patients aged 60 years or older with radiographically confirmed advanced knee osteoarthritis (Kellgren-Lawrence grade III or IV) were eligible and received either a posterior cruciate-retaining (PCR) or posterior-stabilized (PS) prosthesis. Preoperative and postoperative data were collected to evaluate changes in patient-reported outcomes over time. Follow-up evaluations were conducted at six, 12, and 24 months during routine outpatient visits, concluding in October 2024. For patients who missed follow-up appointments, multiple contact attempts were made via telephone and hospital coordination systems to encourage participation.

Exclusion criteria comprised prior revision arthroplasty, bilateral TKA procedures, postoperative complications requiring surgical reintervention, and incomplete follow-up. All participants provided written informed consent for inclusion in the study, application of standardized questionnaires, and longitudinal clinical monitoring.

Surgical procedure and prosthesis characteristics

All total knee arthroplasties were performed under spinal anesthesia using a medial parapatellar approach by the hospital’s orthopedic surgical team. A pneumatic tourniquet was applied prior to incision, with a standardized inflation pressure of 280 mmHg to achieve intraoperative ischemia. Prosthesis selection was based on posterior cruciate ligament (PCL) integrity and intraoperative joint assessment: PCR implants were used when the PCL was intact and permitted balanced flexion-extension gaps. PS designs were selected when the PCL was deficient or when joint stability and kinematic alignment could not be reliably achieved with the native ligament. Both implant types featured a cemented cobalt-chrome femoral component and a titanium tibial baseplate with a modular polyethylene insert. Patellar resurfacing was selectively performed in cases with advanced patellofemoral degeneration. All patients followed a standardized perioperative protocol that included antibiotic prophylaxis with cefazolin (2 g IV) administered 30 minutes before skin incision and repeated every eight hours for the first 24 hours postoperatively, in accordance with institutional guidelines and international recommendations for infection prevention in joint replacement surgery. The protocol also included multimodal analgesia, pharmacologic thromboprophylaxis, and early mobilization under supervised physiotherapy.

Data collection instruments

Patient-reported outcomes were assessed using three Spanish validated instruments applied during follow-up visits at six, 12, and 24 months: the Western Ontario and McMaster Universities Osteoarthritis Index (WOMAC) [6], the 36-Item Short Form Health Survey (SF-36) [7], and the Patient-Reported Outcomes Measurement Information System Global Health (PROMIS-10) [8]. All instruments were administered in person by trained clinical staff and used in their Spanish-language versions, previously validated in Mexican and Latin American populations.

Patient-reported outcomes were assessed using the WOMAC Index, the SF-36 Health Survey, and the PROMIS-10 Global Health questionnaire. The WOMAC is a disease-specific instrument with 24 items evaluating pain, stiffness, and physical function, scored and standardized on a 0-100 scale, where lower scores indicate better clinical status. The SF-36 is a generic quality-of-life instrument with 36 items across eight domains, also scored from 0 to 100, with higher values reflecting better health perception. It provides physical and mental component summary scores. In this study, raw domain scores were used rather than norm-based scoring (NBS), in line with the descriptive nature of the analysis and to maintain consistency with prior orthopedic studies in Latin American populations with limited sample sizes. The PROMIS-10 evaluates global physical and mental health through 10 items, reported as standardized T-scores (mean=50, SD=10), where higher scores denote better self-perceived health. All instruments were applied in their validated Spanish versions.

Statistical analysis

Data analysis was performed using SPSS version 23.0 (IBM Corp., Armonk, NY, USA). Continuous variables were summarized using means and standard deviations (SD), while categorical variables were expressed as absolute frequencies and percentages. Data normality was assessed using the Shapiro-Wilk test to guide appropriate descriptive interpretation. No formal hypothesis testing or inferential statistics were conducted. Instead, trends over time and group differences were descriptively evaluated across follow-up points. The analysis followed an intention-to-describe approach, and all reported results are presented without p-values or effect size estimations.

## Results

A total of 48 patients underwent TKA and were allocated into two groups according to prosthesis design: posterior cruciate-retaining (PCR, n=27) and posterior-stabilized (PS, n=21). The mean age of the study population was 62.1 years, with patients in the PS group being slightly older (64.5 ± 6.1 years) compared to those in the PCR group (60.3 ± 7.7 years). The overall female predominance was evident in both groups, accounting for 66% of the PCR group and 71% of the PS group.

Anthropometric analysis revealed a higher body mass index (BMI) in the PCR group (30.5 ± 5.4 kg/m²) compared to the PS group (29.2 ± 4.4 kg/m²), suggesting a higher prevalence of overweight or obesity in patients receiving PCR prostheses. Surgical characteristics showed that the mean operating time was longer in the PCR group (62.9 ± 14.2 minutes) compared to the PS group (52.3 ± 9.3 minutes). Regarding laterality, right-sided TKA was slightly more frequent in both groups (56% in PCR and 57% in PS), with left-sided procedures accounting for the remaining cases (Table 1).

**Table 1:** Baseline demographic and surgical characteristics by prosthesis type. PCR: posterior cruciate-retaining; PS: posterior-stabilized; SD: standard deviation; BMI: body mass index; min: minutes.

	PCR (n=27)	PS (n=21)
Age, years, mean (SD)	60.3 (7.7)	64.5 (6.1)
Sex, n (%)		
Female	18 (66%)	15 (71%)
Male	9 (34%)	6 (29%)
BMI, kg/m^2^, mean (SD)	30.5 (5.4)	29.2 (4.4)
Operating time, min, mean (SD)	62.9 (14.2)	52.3 (9.3)
Laterality, n (%)		
Right	15 (56%)	12 (57%)
Left	12 (44%)	9 (43%)

**Figure 1:**
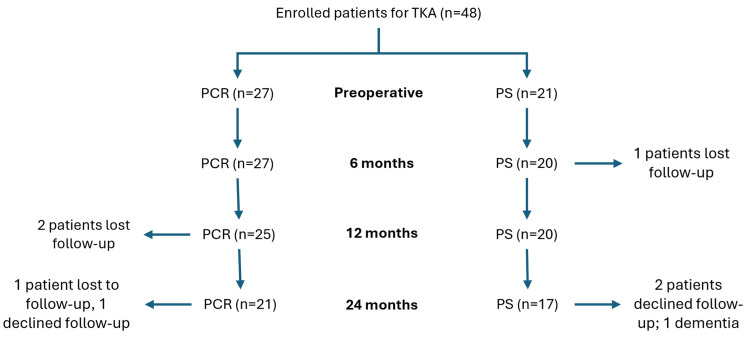
Study flow diagram showing patient follow-up time points. TKA: total knee arthroplasty; PCR: posterior cruciate-retaining; PS: posterior-stabilized.

Patient retention over the follow-up period showed minimal loss. In the PCR group, all 27 patients completed the six-month follow-up, with attrition at subsequent time points due to two patients lost to follow-up by 12 months and an additional three by 24 months (one declined follow-up and one was lost). In the PS group, 20 out of 21 patients completed the six-month follow-up, with further attrition to 17 at 24 months-two declined further follow-up, and one was diagnosed with dementia, limiting long-term evaluation (Figure 1).

At baseline, both groups presented with comparable levels of joint pain, stiffness, and functional limitation as measured by the WOMAC index. The mean preoperative WOMAC pain score was 37.5 ± 3.2 in the PCR group and 34.0 ± 3.1 in the PS group. By six months, both groups showed substantial improvements, with PCR reaching 69.0 ± 3.6 and PS 62.5 ± 4.3. This trend continued through 24 months, where PCR demonstrated superior pain control (77.5 ± 2.0) compared to PS (71.0 ± 1.3). A similar pattern was observed in stiffness and physical function domains, where PCR consistently outperformed PS at each time point. By 24 months, WOMAC Stiffness scores reached 72.5 ± 0.7 (PCR) vs. 62.5 ± 0.9 (PS), and WOMAC physical function scores were 86.5 ± 1.5 vs. 82.6 ± 3.6, respectively.

Health-related quality of life assessed by the SF-36 also showed progressive improvement across both groups. Notably, physical functioning improved from 35.7 ± 10.5 to 66.5 ± 9.5 in PCR and from 41.5 ± 11.2 to 64.1 ± 9.8 in PS. Similarly, role-physical and bodily pain subscales reflected meaningful recovery, with PCR reaching 54.0 ± 8.2 and 72.1 ± 9.6 by 24 months, respectively. Across various domains, including general health, vitality, social functioning, and mental health, both groups experienced improvements. However, PCR consistently achieved slightly higher scores, particularly in the vitality and role-emotional domains.

PROMIS-10 scores supported these findings, reflecting significant gains in both physical and mental health perceptions. The PCR group improved from 38.7 ± 4.6 (physical) and 46.2 ± 5.2 (mental) at baseline to 73.9 ± 4.4 and 75.2 ± 4.6 at 24 months. The PS group also improved, albeit to a lesser degree, ending at 71.3 ± 4.5 (physical) and 72.0 ± 4.6 (mental) by 24 months. These findings collectively indicate that patients with cruciate-retaining prostheses achieved more favorable outcomes in pain relief, joint function, and perceived quality of life than those with posterior-stabilized prostheses throughout the follow-up period (Table 2).

**Table 2:** Descriptive statistics for WOMAC, SF-36, and PROMIS-10 scores across follow-up time points after TKA. WOMAC: Western Ontario and McMaster Universities Osteoarthritis Index; SF-36: 36-Item Short Form Health Survey; PROMIS-10: Patient-Reported Outcomes Measurement Information System Global Health Short Form; SD: standard deviation; TKA: total knee arthroplasty.

	Preoperative		Six months		12 months		24 months	
	PCR (n=27)	PS (n=21)	PCR (n=27)	PS (n=20)	PCR (n=25)	PS (n=20)	PCR (n=21)	PS (n=17)
WOMAC, mean (SD)								
Pain	37.5 ± 3.2	34.0 ± 3.1	69.0 ± 3.6	62.5 ± 4.3	74.5 ± 2.5	68.4 ± 3.9	77.5 ± 2.0	71.0 ± 1.3
Stiffness	31.2 ± 3.6	28.7 ± 2.7	61.3 ± 3.3	52.5 ± 3.2	68.8 ± 2.7	60.0 ± 4.3	72.5 ± 0.7	62.5 ± 0.9
Physical function	67.6 ± 3.7	65.4 ± 4.0	79.4 ± 3.1	78.7 ± 2.1	84.6 ± 1.8	80.6 ± 2.0	86.5 ± 1.5	82.6 ± 3.6
SF-36, mean (SD)								
Physical functioning	35.7 ± 10.5	41.5 ± 11.2	49.3 ± 11.5	54.3 ± 10.5	56.2 ± 12.2	61.0 ± 10.8	66.5 ± 9.5	64.1 ± 9.8
Role-physical	24.3 ± 15.7	33.5 ± 16.4	39.6 ± 15.8	42.3 ± 13.8	45.4 ± 12.5	44.6 ± 13.0	54.0 ± 8.2	51.0 ± 10.5
Bodily pain	28.5 ± 13.5	39.8 ± 13.2	47.3 ± 12.7	52.9 ± 4.0	69.8 ± 10.2	66.5 ± 10.0	72.1 ± 9.6	70.0 ± 9.4
General health	44.2 ± 12.7	48.5 ± 12.2	49.9 ± 11.4	51.6 ± 4.0	62.7 ± 12.5	65.0 ± 10.0	67.0 ± 9.5	66.2 ± 9.2
Vitality (energy)	40.0 ± 12.1	43.1 ± 7.4	50.6 ± 8.0	52.2 ± 6.0	60.2 ± 10.0	58.6 ± 9.8	63.0 ± 6.2	60.1 ± 10.3
Social functioning	57.5 ± 13.0	56.3 ± 13.2	59.3 ± 12.2	57.5 ± 11.0	61.1 ± 11.8	60.5 ± 11.5	65.2 ± 10.8	63.4 ± 10.5
Role-emotional	55.0 ± 14.0	56.0 ± 14.2	58.2 ± 13.5	58.0 ± 4.6	63.5 ± 12.5	64.8 ± 12.3	68.0 ± 11.5	66.0 ± 11.0
Mental health	60.0 ± 12.0	60.2 ± 11.8	63.5 ± 11.5	62.0 ± 6.2	68.8 ± 10.2	67.0 ± 10.0	69.3 ± 9.8	68.0 ± 9.5
PROMIS 10, mean (SD)								
Physical	38.7 ± 4.6	38.4 ± 3.5	53.0 ± 4.8	52.4 ± 4.7	63.4 ± 7.5	62.9 ± 8.6	73.9 ± 4.4	71.3 ± 4.5
Mental	46.2 ± 5.2	46.0 ± 5.1	56.5 ± 5.0	56.3 ± 5.0	66.8 ± 6.8	66.6 ± 3.9	75.2 ± 4.6	72.0 ± 4.6

## Discussion

TKA remains the cornerstone for restoring function and improving the quality of life in patients with advanced knee osteoarthritis. Although its overall clinical efficacy is well established, postoperative recovery trajectories may vary according to prosthesis design [9]. Demographically, our cohort aligned with global TKA populations while exhibiting regional distinctions. The mean age was 60.3 years in the PCR group and 64.5 years in the PS group, which is notably younger than the averages reported in high-volume registries, such as Weber et al., where most patients exceed 70 years. This may be due to the earlier disease onset associated with prevalent metabolic comorbidities in the Mexican population [10]. The female predominance (66% in PCR; 71% in PS) mirrored global epidemiologic patterns of osteoarthritis. Laterality was balanced, with a slight predominance of right-sided procedures, in line with national surgical trends [11]. BMI averaged 30.5 kg/m² in the PCR group and 29.2 kg/m² in the PS group, categorizing most patients as overweight or obese, a relevant finding given the established association between elevated BMI, earlier symptom onset, impaired postoperative recovery, and increased complication rates [12]. These observations are consistent with findings from Li et al., who identified high BMI as a key predictor of variability in TKA outcomes [13]. Notably, operative times were longer for PCR procedures, consistent with prior reports attributing the difference to the technical challenges of preserving and balancing the posterior cruciate ligament [14].

Functionally, both groups experienced improvement over time, yet PCR patients showed greater and more sustained gains. WOMAC outcomes demonstrated superior pain control, stiffness reduction, and physical function in the PCR group, particularly beyond 12 months. This contrasts with the findings of van den Boom et al., who reported no significant differences in WOMAC domains during the first postoperative year [15]. Their limited follow-up may have missed the late divergence observed in our cohort. Additionally, Rampazo-Lacativa et al. reported high responsiveness in WOMAC pain and function domains but noted ceiling effects at two years in well-recovered patients, an effect mirrored in our high-scoring PCR subgroup. However, this limitation was attenuated through the use of complementary instruments [16].

SF-36 scores further supported the functional advantage of PCR prostheses, with greater improvements observed in physical functioning, role-physical, and bodily pain domains throughout follow-up. These findings are consistent with the results reported by Escobar et al., where the most notable postoperative gains following TKA occurred precisely in those domains, with changes exceeding 18 points during the first six months and remaining stable at two years. In our cohort, PCR patients also demonstrated meaningful increases in vitality and role-emotional scores, dimensions in which Escobar et al. found only modest improvements. This contrast may reflect differences in prosthesis design or rehabilitation dynamics and underscores the potential broader impact of cruciate retention on both physical and psychosocial recovery [17]. Overall, our results reaffirm the value of SF-36 as a sensitive instrument to capture functional recovery profiles in TKA recipients.

PROMIS-10 added critical value by capturing global perceptions of physical and mental health. PCR patients showed higher scores in both domains by 24 months (physical: 73.9 vs. 71.3; mental: 75.2 vs. 72.0), consistent with findings from comparative studies evaluating PROMIS against tools like EQ-5D and Oxford Knee Score (OKS). Unlike WOMAC or SF-36, PROMIS-10 demonstrated no ceiling effects and offered broader construct coverage, confirming its utility in evaluating patient-centered recovery, especially in high-functioning individuals [18].

Several limitations should be acknowledged. The sample size restricted the ability to perform multivariate or association analyses. Prosthesis allocation was based on intraoperative criteria rather than randomization, which may introduce allocation bias. Although perioperative protocols were standardized, rehabilitation adherence was not systematically measured. Additionally, the potential for ceiling effects in WOMAC at later follow-up points could have reduced sensitivity to detect further improvement in high-functioning individuals; however, the inclusion of SF-36 and PROMIS-10 helped address this limitation. Finally, range of motion was not consistently recorded across follow-up visits, which limited our ability to correlate objective functional measures with patient-reported outcomes.

## Conclusions

Throughout the 24-month follow-up, patients who received PCR prostheses consistently reported higher functional recovery and quality of life scores compared to those with PS prostheses. WOMAC scores revealed greater improvements in pain, stiffness, and physical function in the PCR group. Similarly, SF-36 indicated better physical functioning, vitality, and emotional role scores, while PROMIS-10 reflected higher global health perception. Although the study was descriptive, the consistency of these findings suggests a favorable trend for PCR prostheses. Further multicenter studies with standardized rehabilitation protocols and larger samples are warranted to confirm these observations.
